# Nondetrimental impact of two concomitant entomopathogenic fungi on life history parameters of a generalist predator, *Coccinella septempunctata* (Coleoptera: Coccinellidae)

**DOI:** 10.1038/s41598-021-00037-8

**Published:** 2021-10-19

**Authors:** Muhammad Rizwan, Bilal Atta, Muhammad Arshad, Rashad Rasool Khan, Asli Dageri, Misbah Rizwan, Muhammad Irfan Ullah

**Affiliations:** 1 Rice Research Institute, Kala Shah Kaku, Sheikhupura, Pakistan; 2 Department of Entomology, University of Sargodha, Sargodha, 40100 Pakistan; 3 China-Pakistan Joint Research Centre for Citrus Disease and Insect Pest Management, University of Sargodha, Sargodha, 40100 Pakistan; 4 Department of Entomology, University of Agriculture, Faisalabad, Pakistan; 5 Department of Molecular Biology and Genetics, Necmettin Erbakan University, 42090 Meram, Konya Turkey

**Keywords:** Entomology, Microbiology

## Abstract

The non-persistent impact of biocontrol agents can be revealed for pest control when associated entomopathogenic fungi (EPFs) negatively affect the natural enemies. In this assay, impacts of *Beauvaria bassiana* (Balsamo) Vuillemin, and *Metarhizium anisopliae* (Metschnikoff) Sorokin were studied for their compatibility or side effects on life table parameters of an important generalist predator, *Coccinella septempunctata* L. The results indicated non-significant impacts of both EPFs on life table parameters of *C. septempunctata*. The development time (egg-adult) was not significantly different in control (69.79 days) and EPFs treated *C. septempunctata* (69.35–80.07 days). Both fungi did not induce any significant changes in the fecundity, adult pre-oviposition period (APOP), total preoviposition period (TPOP), and mean generation time (T) as compared to control treatment. Similarly, no difference in fecundity rate of *C. septempunctata* was observed after EPFs treatment (287.7–288.5) compared to control (290.0). The highest net reproductive rate (*R*_*0*_) occurred in control (87.05 offspring individual^−1^) and *M. anisopliae* (86.31 offspring individual^−1^) as compared to *B. bassiana* treated beetles (76.97 offspring individual^−1^). The age-specific fecundity curves indicated that the *C. septempunctata* had a similar fecundity rate in both EPFs treatments and control. This study demonstrates no significant side effects of *B. bassiana* and *M. anispoliae* on the performance and biology of *C. septempunctata*. Considering the compatibility of both EPFs with *C. septempunctata*, their combinations can be recommended in various integrated pest management programs.

## Introduction

Pesticide applications are yet the priority of farmers and pest exterminators for quick pest control and effective crop production^1,2^. However, negative impacts of injudicious pesticides applications are widely reported on human health and the environment^3,4^ as well as non-target organisms including natural enemies of insects^5,6^. Besides resistance development in insects^7^, insecticides have a suppressive impact on biocontrol agents^8^ notably through direct mortality of exposed arthropods as well as multiple sublethal physiological (neurobiology, development, adult longevity, immunology, fecundity, and sex ratio) and behavioral side effects (mobility, navigation, feeding, oviposition, and learning performance)^5^. They can also drive habitat changes, induce hormesis effects in key pests and modulate direct and indirect interactions among species within food webs^9–13^. Alternatives of chemical pesticides are under investigation for reducing the chemical load on the environment and mitigating pollution. The role of natural enemies including the predators, parasites/parasitoids, and pathogens is widely reported for regulation of insect populations in both pest and non-pest insects’ context^14^. In field conditions, insect pests may be utilized by more than one natural enemy. Thus, these natural enemies interact with each other and with their prey/host at the same time^15^, however, the impact on the prey/host can be either additive, antagonistic, or synergistic^16^.

Ladybird beetle, *Coccinella septempunctata* L. (Col., Coccinellidae) is a common generalist predator of soft-bodied agricultural insect pests^17^. It is the most common predator found in the agricultural ecosystem and plays a key role in controlling many insect pests. Immature larval instars are comparatively less voracious, and the feeding rate increases tremendously in the case of later stages as well as adults^18^. *Coccinella septempunctata* tends to feed on various aphid species^19^, however, its development and biological parameters such as survival, and fecundity, etc. depend upon the prey it feeds^20^.

Considerable advancements are reported in the field of insect pathology and the role of entomopathogenic microorganisms is widely investigated for effective pest control under controlled and open field conditions^14^. Currently, biopesticides are being used for insect pest management due to less hazardous impact on the environment, beneficial fauna, and ultimately human health^21,22^. Entomopathogenic fungi (EPFs) have been used for the management of insect pests ranging from stored grain pest management^23^, laboratory testing^22^, greenhouse assay^21^, and field trials^24^. However, studies regarding the impact of these biopesticides including EPFs on the biological control agents naturally present in the field are still a researchable topic.

EPFs have been reported as an effective tool in controlling a broad range of insect pests^21,22,25,26^. High virulence makes the EPFs a useful strategy against insect pests. However, the success of EPFs is also associated with non-virulence/compatibility to biocontrol agents like predators and parasitoids, which should be tested before recommending to the farmers. *Beauvaria bassiana* (Balsamo) Vuillemin (Ascomycota: Hypocreales) is being used successfully against various insect pests such as *Cnaphalocrocis medinalis*^21^, *Nilaparvata lugens*^22^, and *Tribolium castaneum*^23^, *Helicoverpa armigera*^27^, *Plutella xylostella*^28^*, Cosmopolites sordidus*^29^, aphid species^30^, and many stored grain insect pests^26^. *Metarhizium anisopliae* (Metschnikoff) Sorokin (Hypoc., Clavicipitaceae) is another fungus that naturally grows in soil worldwide^31^ and plays a key role as an active biological control agent for different insect pests^32^.

*Beauvaria bassiana* and *M. anisopliae* are being considered and used throughout the world as strong and effective biological control agents^33^. However, the success of these fungal species as biocontrol agents does not depend only on the selective and effective killing of insect pests but also on very few or no adverse effects against beneficial non-target insects present in the field. Due to the high importance of coccinellid beetles in aphid biological control programs, it is necessary to determine the impact of other biological agents such as EPFs on these generalist predators. Although studies are available on the interaction of EPFs with other biological control agents^34,35^, their effect on the life table and demographic parameters on the coccinellids is limited. Life table is an important tool to study population ecology and summarize the survival and reproductive potential of insect populations on different hosts^36–38^. The age-stage, two-sex life tables show an improvement over traditional life tables that are incapable of describing the important feature of stage differentiation and ignore the male population^39,40^. On the other hand, age-stage, two-sex life tables consider male individuals and can calculate stage differentiation (i.e. metamorphosis)^41,42^. Furthermore, it can accurately determine the actual life history of the insect species and is useful to study various ecological aspects of insect pests and their associated natural enemies^43,44^. In this bioassay, we have studied the effect of the two EPFs (*B. bassiana* and *M. anisopliae*) on the life table and demographic parameters of the ladybird beetle (*C. septempunctata*).

## Methods

### *Coccinella septempunctata* culture

Adults of ladybird beetle, *C. septempunctata*, were collected from field research area (31.7213° N, 74.2700° E) of Rice Research Institute, Kala Shah Kaku, Punjab, Pakistan. Adults were maintained in plastic jars (20 × 15 cm) with an abundant supply of aphids to obtain eggs. Eggs were transferred to Petri dishes and the newly emerged larvae were supplied with nymphs of *Aphis craccivora* Koch (Hem., Aphididae). Aphids were also reared on wheat (*Triticum aestivum* L.) plants in the laboratory for abundant supply as a feed for coccinellid beetles. Coccinellid beetle was reared up to the 4th generation to use in the experiment.

### Entomopathogenic fungi

Both entomopathogenic fungi, *B. bassiana* and *M. anisopliae* were obtained from AgriLife SOM Phytopharma (India) Limited (http://www.agrilife.in) in talc form and were tested at 1 × 10^8^ CFU/ml recommended by the manufacturer to control various insect pests. Both fungi have been reported effective in controlling insect pests such as *Diaphorina citri* Kuwayama (Hem., Liviidae)^45^, *Cnaphalocrocis medinalis* (Guenee) (Lep., Pyralidae)^21^, and *Nilaparvata Lugens* (Stål) (Hom., Delphacidae)^22^. The quality of conidia was determined by counting the conidial concentration in a Neubauer chamber, hemacytometer. The germination of conidia was determined on Potato dextrose agar (PDA) based on the counts of 200 random conidia per plate, 18 h post-incubation at 25 ± 2 °C^46^, and the suspensions with 90% conidial germination were used in the bioassay.

### Life table study

To study the pre-imaginal development and survival, 60 eggs of beetles were obtained from the reared culture for each treatment (2 EPFs and control) and placed separately in clean Petri plates. The egg incubation period was recorded at 12 h intervals. Newly molted 2nd instar larvae of coccinellid beetle were treated with EPFs and distilled water was used in the control group. Sprayed larvae were shifted into new glass Petri dishes, and were kept without food for 3 h to avoid EPFs ingestion through food material. Data for the duration of each developmental stage from larvae to adults were recorded at 12 h intervals. Adults were selected from the corresponding treatment with the immature stages and were sexed to record the longevity and fecundity rate till the death of adults. The adult pairs were kept in a clean plastic jars and provided aphids daily. The laid eggs were separated daily for each couple. The experiment was performed under controlled conditions of 25 ± 2 °C temperature, 65 ± 5% R.H. and 16:8 h (L:D) photoperiod.

### Life table analysis

Using raw data, the stage mean, age-stage–specific survival rate (*s*_*xj*_), age-stage reproductive value (*v*_*xj*_), age-stage–specific fecundity (*f*_*xj*_), age-stage life expectancy (*e*_*xj*_), age-specific survival rate (*l*_*x*_), age-specific fecundity (*m*_*x*_), age-specific net maternity (*l*_*x*_*m*_*x*_), and life table parameters (*R*_*0*_, net reproductive rate; *r*, intrinsic rate of increase; *λ*, finite rate of increase; and T, mean generation time) were calculated using TWOSEX-MSChart program^47^. The quick paired bootstrapping (Paired 1 by 1) technique with 100,000 replications was used to minimize the variation in the results for calculating the mean and standard error of the population^48^ by TWOSEX-MSChart program^47^. The statistical significance of the observed differences between three treatments was evaluated by TWOSEX-MSChart software.

The age-specific survival rate (*l*_*x*_, *m*_*x,*_ and *R*_*0*_) was calculated as:

$$l_{x} = \mathop \sum \limits_{j = 1}^{k} S_{xj}$$

$$m_{x} = \frac{{\mathop \sum \nolimits_{j = 1}^{k} S_{xj} f_{xj} }}{{\mathop \sum \nolimits_{j = 1}^{k} S_{xj} }}$$

$$R_{0} = \mathop \sum \limits_{x = 0}^{\infty } l_{x} m_{x}$$

where k denotes the number of stages, *x* = age in days, *j* = stage, *R*_*0*_ (net reproductive rate) is the average number of offspring per female during its whole life cycle.

The intrinsic rate of increase (*r*), finite rate of increase (*λ*), and mean generation time (*T*) is calculated as:

$$\mathop \sum \limits_{x = 0}^{\infty } e^{ - r(x + l)} l_{x} m_{x} = 1$$

$$\lambda = e^{r}$$

$$T = \ln Ro/r$$

The life expectancy (*e*_*xj*_) is referred to as the expected life of an individual of age *x* and stage *j* is calculated by the equation suggested by Chi and Su^44^:

$$e_{xj} = \mathop \sum \limits_{i = x}^{\infty } \mathop \sum \limits_{y = j}^{k} \user2{ S}_{iy}^{\user2{^{\prime}}}$$

where ${\varvec{S}}_{iy}^{\user2{^{\prime}}}$ is the probability that individuals of age *x* and stage *j* will survive to age *i* and stage *y*, and is calculated by assuming ***S***_*xj*_ = 1.

The reproductive value (*v*_*xj*_) was calculated by the equation suggested by Tuan et al.^49^:

$$v_{xj} = \frac{{e^{r(x + 1)} }}{{S_{xj} }}\mathop \sum \limits_{i = x}^{\infty } e^{ - r(i + 1)} \mathop \sum \limits_{y = j}^{\beta } \user2{S^{\prime}}_{iy} f_{iy}$$

The projection of the population growth of *C. septumpunctata* was calculated through Timing-MSChart^47^. The population growth was simulated for an initial population of 10 eggs over a period of 60 days.

## Results

Entomopathogenic fungi lasted no significant impact on the duration of different life stages of the coccinellid beetle when compared to the control group (*P* > 0.05*,* Supplementary Table S1). *Coccinella septumpunctata* larvae infected with the two fungi (*B. basiana* and *M. anisopilae*) possessed the same larval duration (4.25–4.66 d and 4.21–4.61 d, respectively) and no significant difference (*P* > 0.05) was noticed with those of the control (4.25–4.68 d). Similarly, the pupal period was recorded as 5.18 and 5.08 d when larvae were infected with *B. basiana* and *M. anisopilae*, respectively as compared to the control group (5.17 d). Adults who emerged from EPFs-infected immature stages survived for 40.2 to 41.2 d. while adults’ longevity from the control group was 40.6 d. Female adults lived more days than males in both EPF treatment and control (Table 1).

**Table 1:** Developmental period (days) of *Coccinella septumpunctata* after exposure of entomopathogenic fungi.

Life stages	N	*B. bassiana*	Development time (mean ± SE)			
			N	*M. anisopliae*	N	Control
Egg	60	5.92 ± 0.03 a	60	5.95 ± 0.03 a	60	5.92 ± 0.04 a
L1	52	4.25 ± 0.06 a	55	4.21 ± 0.06 a	53	4.25 ± 0.06 a
L2	45	4.66 ± 0.07 a	46	4.61 ± 0.07 a	44	4.68 ± 0.07 a
L3	39	4.55 ± 0.08 a	38	4.50 ± 0.07 a	38	4.58 ± 0.08 a
L4	32	4.59 ± 0.09 a	29	4.52 ± 0.08a	29	4.59 ± 0.09 a
Pupa	28	5.18 ± 0.07 a	24	5.08 ± 0.09 a	29	5.17 ± 0.07 a
Adult Longevity		40.2 ± 3.62 a		41.2 ± 1.24 a		40.6 ± 1.17 a
Female	18	45.1 ± 0.59 a	16	45.1 ± 0.67 a	18	45.1 ± 0.62 a
Male	10	33.3 ± 0.75 a	8	33.5 ± 0.65 a	11	33.2 ± 0.65 a

SE was estimated by Bootstrapping (100,000 replications), L1–L4 indicate the larval instar, means sharing similar letters in each row are not significantly different at *P* > 0.05, N = numbers of individual *C. septumpunctata* that completed a stage.

Population parameters (*R*_*0*_*,* T*, r, λ*) of *C. septumpunctata* recorded in treated and control groups are displayed in Table 2. Not only APOP, TPOP, and oviposition period remained unaffected by the application of EPFs, fecundity rate of coccinellid beetle also remained unchanged (*P* > 0.05, Supplementary Table S2) in EFP-treated (287.7–288.5 eggs) and control group (290.0 eggs). Net reproductive rate (*R*_*0*_), was significantly affected (*P* < 0.05) by *B. basiana* (76.97 offspring/individual), while no significant difference (*P* = 0.6791) was recorded in *M. anisopilae* treated (86.31 offspring/individual) and control group (87.05 offspring/individual). In comparison to control treatment, no significant difference (*P* > 0.05) was recorded in mean generation time (T), intrinsic rate of increase (*r*), and finite rate of increase (*λ*) when larvae were infected with EPFs (Table 2).

**Table 2:** Comparison of reproductive and life table parameters (mean ± SE) of *Coccinella septumpunctata* after exposure of entomopathogenic fungi.

Parameters	*B. bassiana*	*M. anisopliae*	Control
APOP (d)	5.190 ± 0.34 a	5.170 ± 0.32 a	5.140 ± 0.34 a
TPOP (d)	32.12 ± 0.11 a	32.94 ± 0.57 a	33.33 ± 0.61 a
Oviposition (d)	13.00 ± 0.41 a	13.22 ± 0.38 a	13.06 ± 0.37 a
Fecundity	288.5 ± 13.8 a	287.7 ± 12.1 a	290.0 ± 12.0 a
*R*_*0*_ (offspring individual^−1^)	76.97 ± 16.9 b	86.31 ± 17.4 a	87.05 ± 17.4 a
T (d)	45.31 ± 0.49 a	46.34 ± 0.69 a	46.39 ± 0.69 a
*r* (d^−1^)	0.095 ± 0.01 a	0.095 ± 0.01 a	0.095 ± 0.02 a
*λ* (d^−1^)	1.100 ± 0.01 a	1.101 ± 0.01 a	1.101 ± 0.01 a

SE was estimated by bootstrapping (100,000). Whereas APOP, TPOP, T, r, λ, and Ro represents an adult pre-oviposition period (days), total pre-oviposition period (days), mean generation time (days), intrinsic rate of increase (d^−1^), and finite rate of increase (d^−1^), net reproductive rate (offspring) respectively, means sharing similar letters in each row are not significantly different at *P* > 0.05.

**Figure 1:**
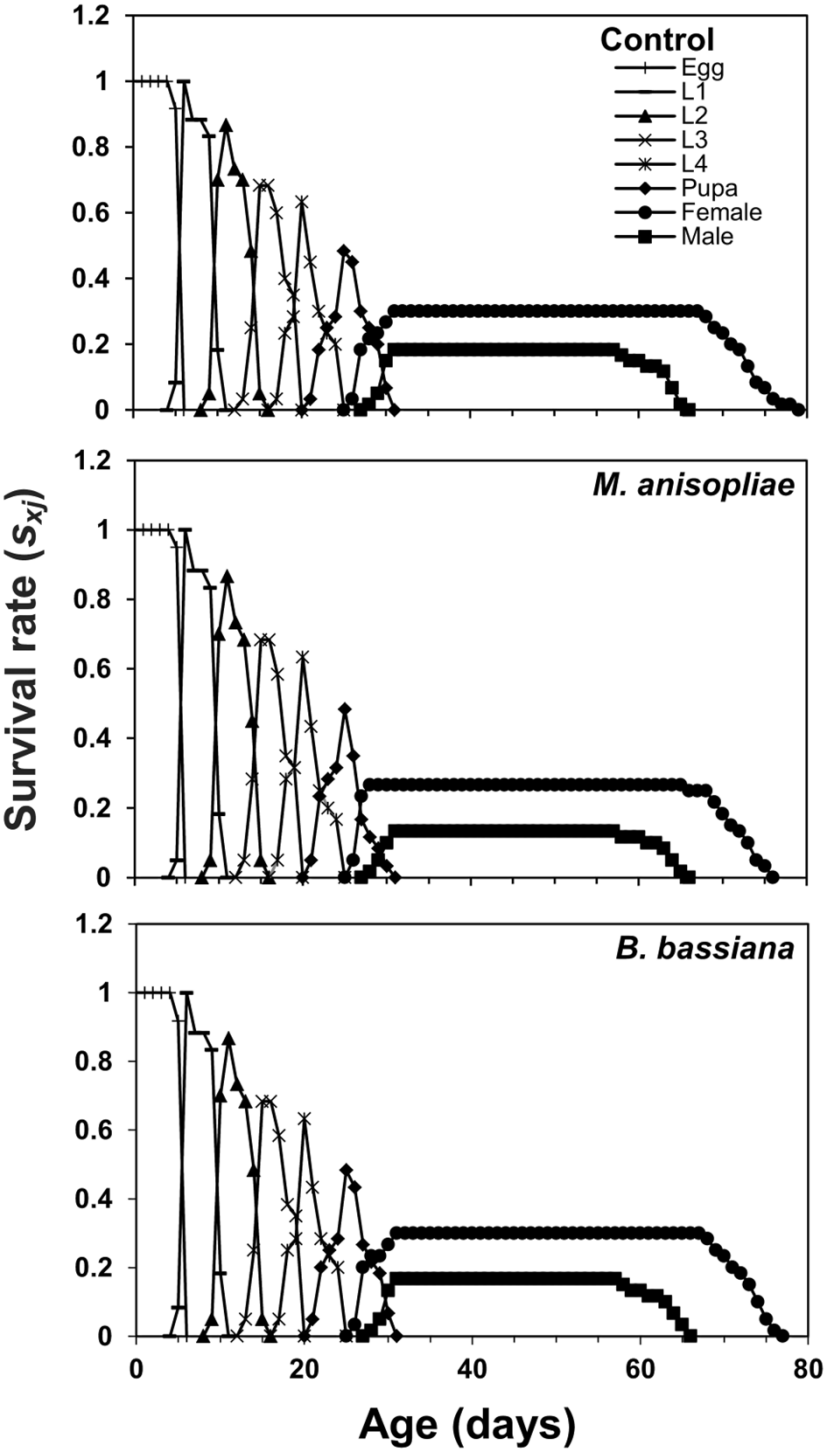
Age-stage–specific survival rate (*s*_*xj*_) of *Coccinella septumpunctata* after exposure of entomopathogenic fungi.

**Figure 2:**
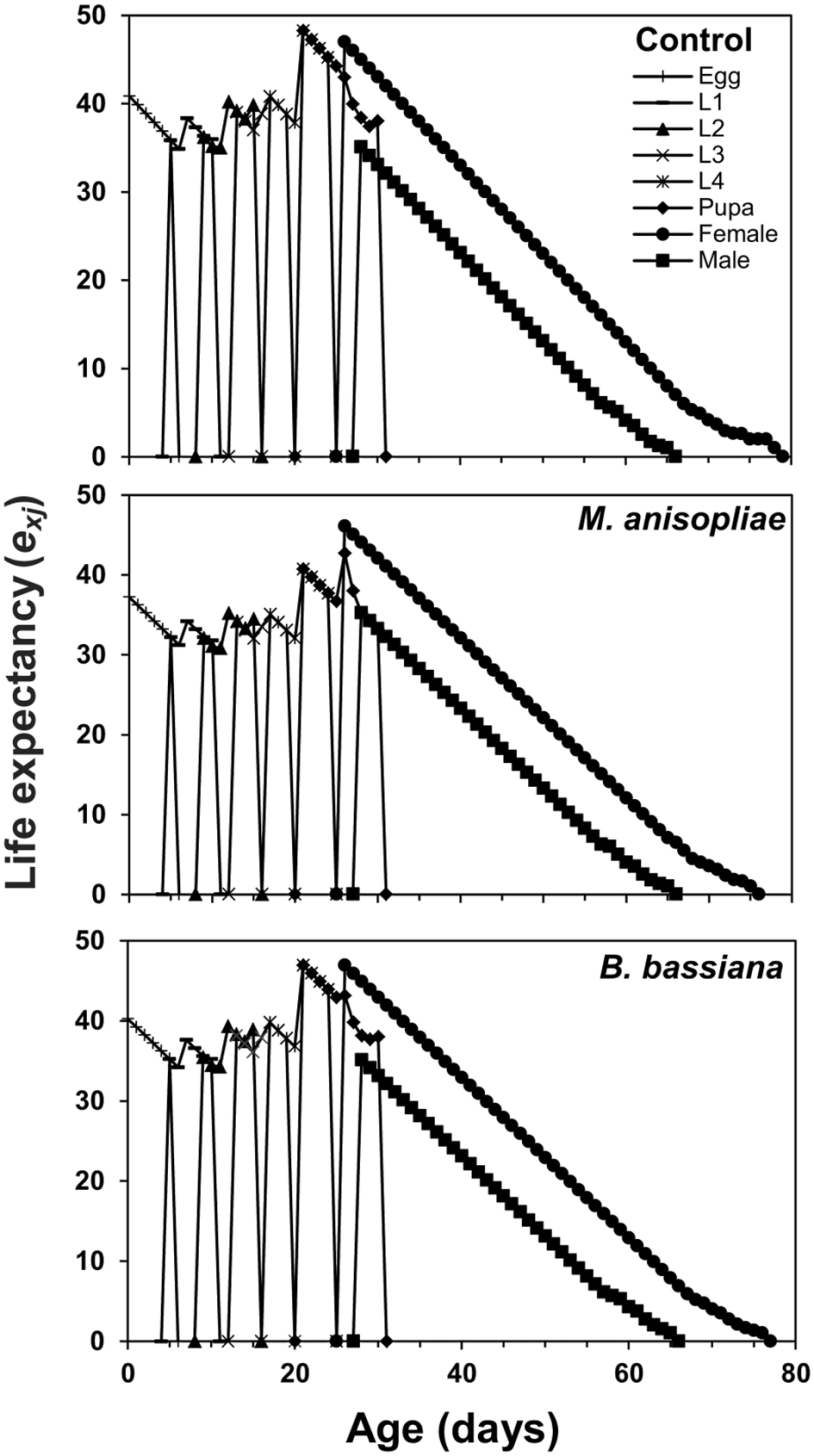
Age-stage–specific life expectancy (*e*_*xj*_) of *Coccinella septumpunctata* after exposure of entomopathogenic fungi.

**Figure 3:**
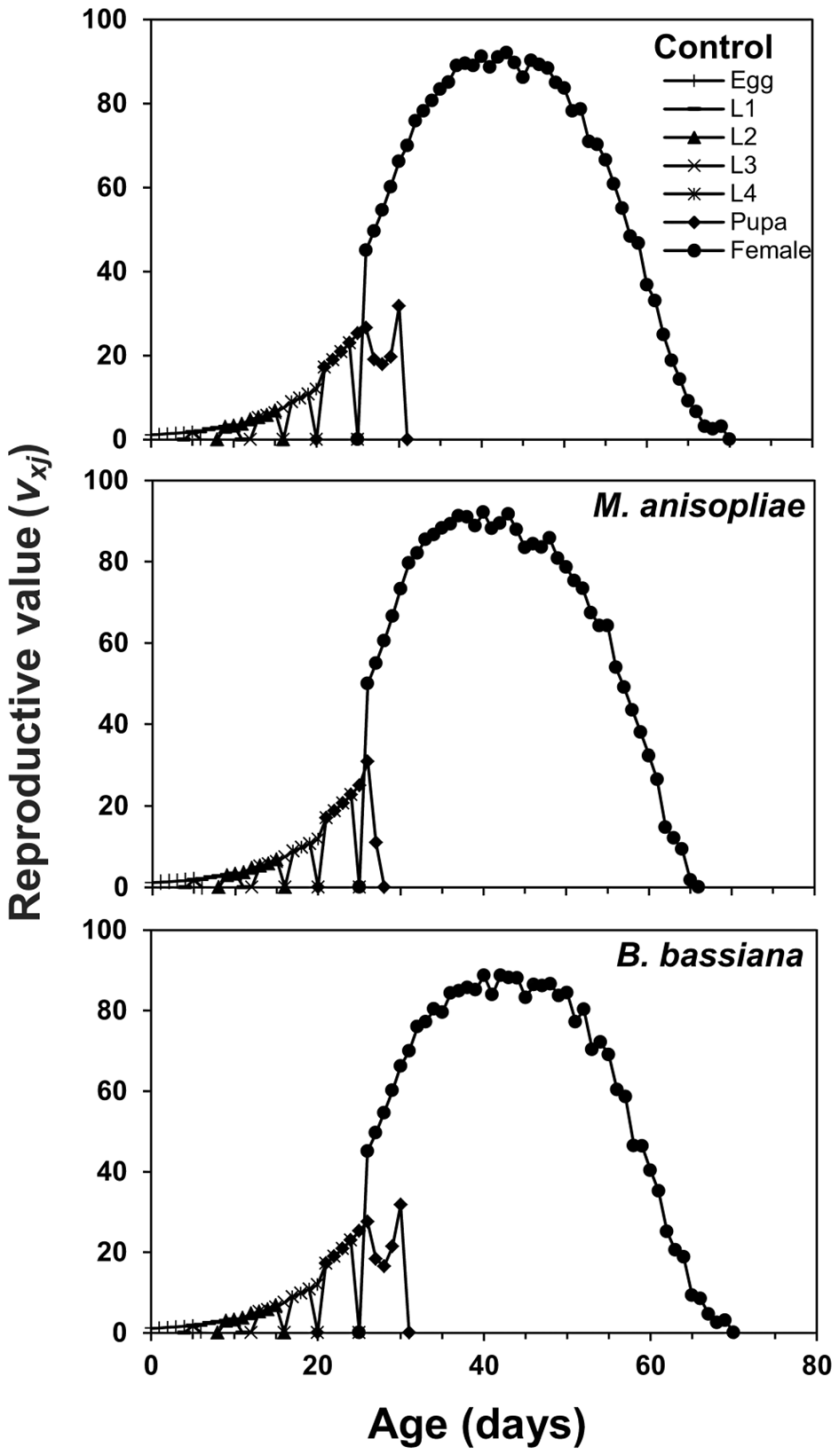
Age-stage–specific reproductive rate (*v*_*xj*_) of *Coccinella septumpunctata* after exposure of entomopathogenic fungi.

**Figure 4:**
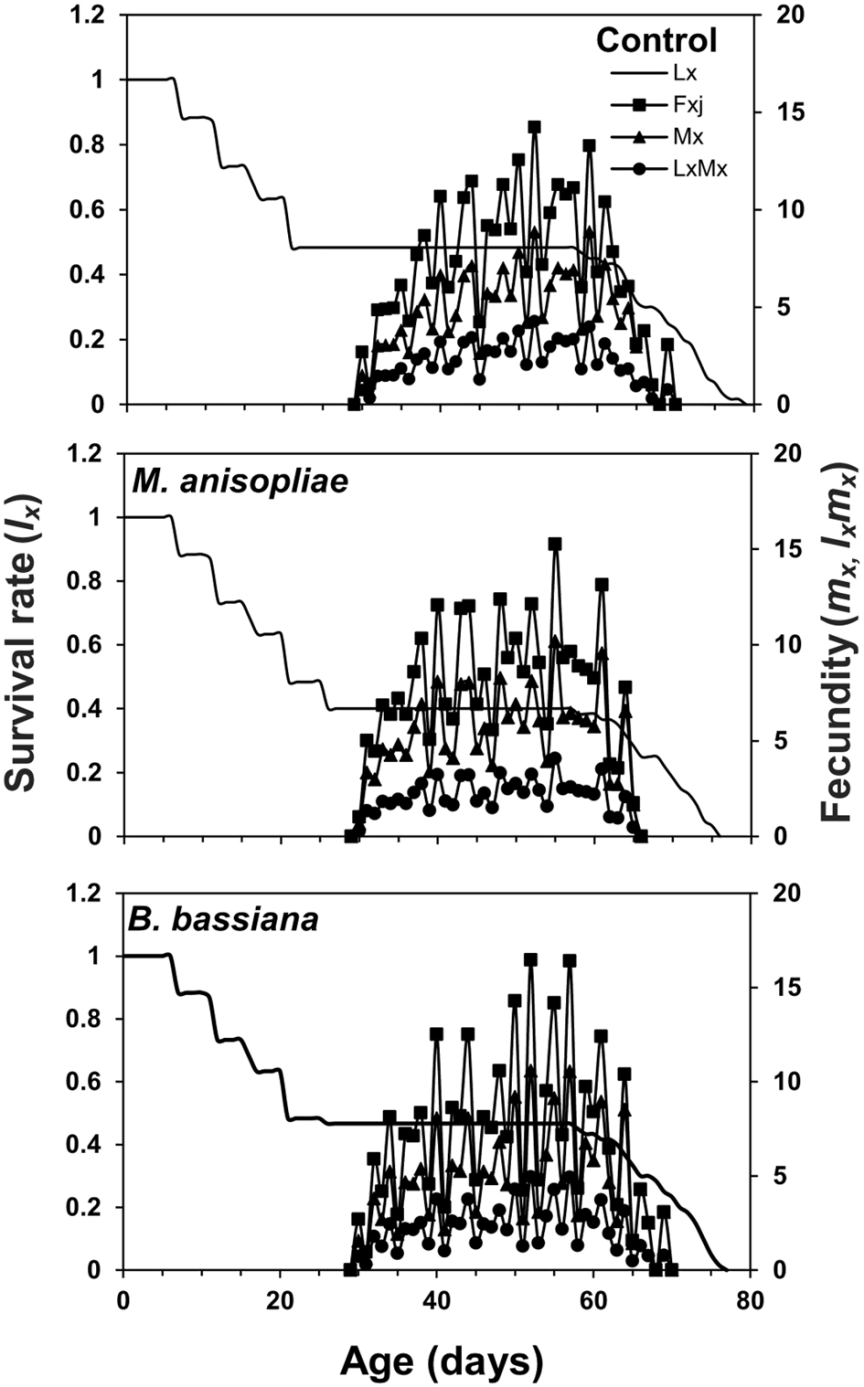
Age-specific survival rate (*l*_*x*_), age-stage–specific fecundity (*f*_*xj*_), age-specific fecundity (*m*_*x*_), and age-specific maternity (*l*_*x*_*m*_*x*_) of *Coccinella septumpunctata* after exposure of entomopathogenic fungi.

**Figure 5:**
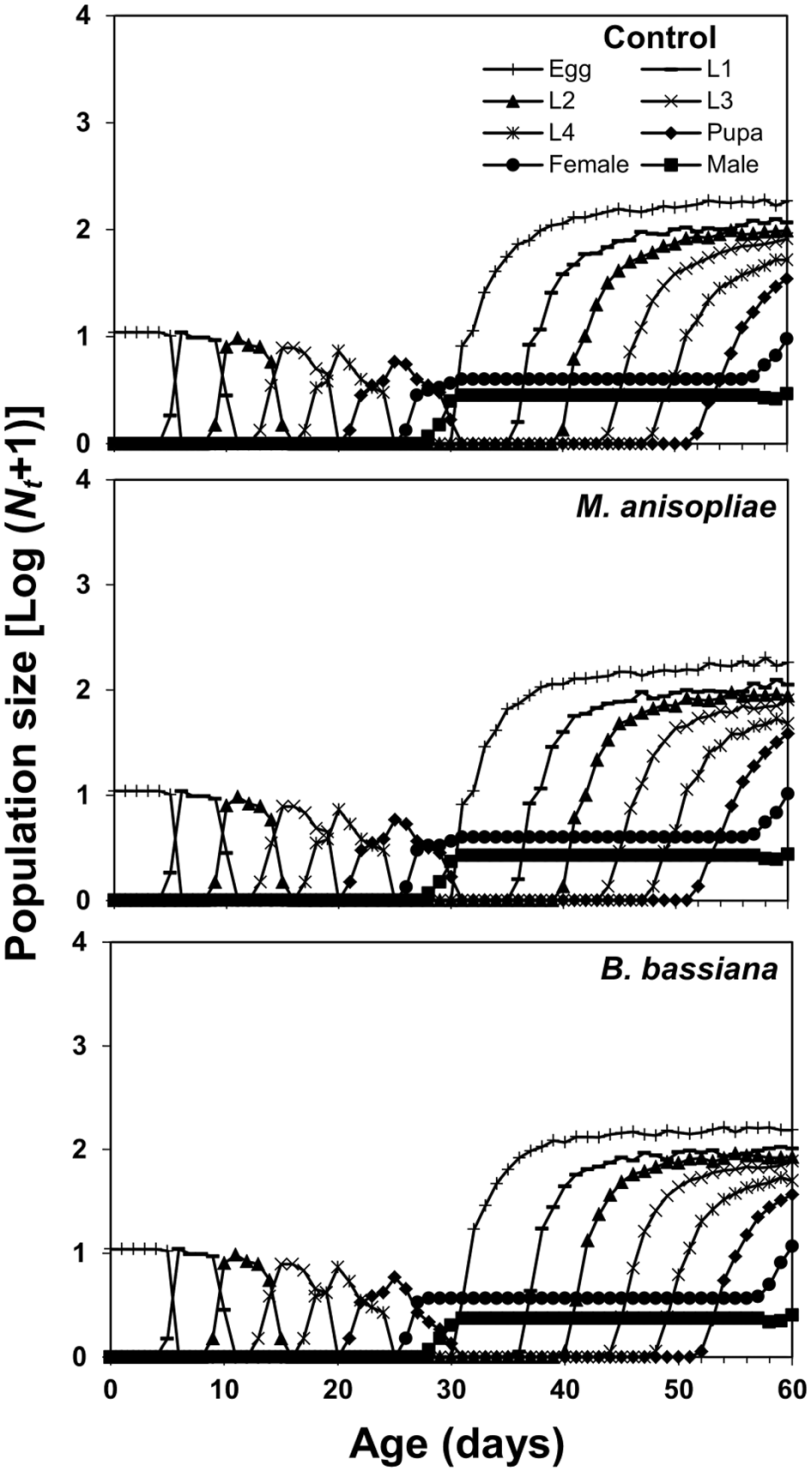
Population projection showing the change of stage-structure of *Coccinella septempunctata* after exposure of entomopathogenic fungi.

The age-stage survival curve (*s*_*xj*_) showed a higher survival rate of coccinellid beetles in control and *B. bassiana* treatment as compared to *M. anisopliae*. Surviving trends of 60, 51, 29, 29 individuals in control, 60, 51, 29, 28 individuals in *B. bassiana* and 60, 52, 29, 24 individuals in *M. anisopliae* treatment were recorded at ages of 1, 10, 20, and 30 days respectively (Fig. 1). The age-stage specific life expectancy curve (*e*_*xj*_) describes that newly born larvae of coccinellid beetle may live for 40.87, 40.22, and 37.22 days in control, *B. bassiana*, and *M. anisopliae* treatments, respectively. The female adults were expected to live longer than male adults and were estimated to survive for 47.00 days in control, 46.89 days in *B. bassiana*, and 46.06 days in *M. anisopliae* treatment. Males, on the other hand, were estimated to live for 35.09 days in control, 35.10 days in *B. bassiana*, and 35.25 days in *M. anisopliae* treatment (Fig. 2). The highest age-stage specific reproductive rate (*v*_*xj*_) value for females was recorded as 92.07 at 43rd day in control, 88.68 at 42nd day in *B. bassiana*, and 91.67 at 43rd day in *M. anisopliae* treatments (Fig. 3). The *l*_*x*_, *f*_*xj*_, and *m*_*x*_ curves indicated that the coccinellid beetle had a similar survival rate and fecundity in both EPFs treatments and control. The *f*_*xj*_ curve indicates age-stage-specific female fecundity of a female and the following trend of maximum egg-laying was found; 15.67 eggs at the age-stage of 52nd day in control, 15.66 eggs at the age-stage of 55th day in *M. anisopliae*, and 14.84 egg at the age-stage of 52nd day in *B. bassiana* (Fig. 4). The population projection reveals the projected growth of an insect over a specific period under the same conditions. In this analysis, we have calculated the population of *C. septempunctata* for 60 days. The initials size of the population was recorded 10 eggs for each treatment. The highest population size of beetle (573.43 individuals) was in the control group followed by 553.21 individuals in *M. anisopliae* treatment. The total population size in *B. bassiana* treatment was 510.46 individuals (Fig. 5).

## Discussion

Interaction between natural enemy species of an organism present in the field is one of the most important factors to determine the biological control programs^50^. Various studies have been performed on interactions between EPFs and other biological control agents (parasitoids and predators)^34,51,52^; however, little information is available about the effects of EPFs on the demography of coccinellid species. Even though the EPFs and coccinellids may occupy the same habitat, it is important to understand their interspecific interactions during coexistence. In the present study, we investigated the biological parameters of *C. septempunctata* beetles infected with two EPFs (*B. basiana* and *M. anisopilae*). The effects were shown through surface contact of EPFs to newly emerged larvae of the generalist predator. Such information could be useful to assess the compatibility of this coccinellid beetle with EPFs in IPM programs of aphid species. Using EPFs in pest control programs, their selection is very important to obtain effective control of agricultural pests^53^. The compatibility of EPFs with other biocontrol agents could be useful to achieve higher pest control by decreasing the application of synthetic insecticides and minimizing the resistance to insecticide^54^.

Our findings showed that the life span of the coccinellid beetle remained unaffected when infected with both EPFs, as no significant difference was noticed in the developmental period of each larval stage, pupa, and adult beetles when compared to control treatment. Both fungi were found to have a negligible threat on this coccinellid beetle and hence their recommended concentrations are suggested as compatible with coccinellid predators. In general, the safety of EPFs to non-target organisms has been reported earlier^55,56^. Zaki^57^ and Ormond et al.^52^ documented that *B. bassiana* did not affect the developmental period of *Coccinella* spp. The larval mortality was noticed in both the treatments as well as control in the reported experiment, however, it may be attributed to natural death because all grubs of beetle did not reach adulthood. However, EPFs might have a negative effect on newly emerged larvae of predators. As reported by Sayed et al.^58^, the first larval instar of *C. undecimpunctata* was affected negatively by *B. bassiana*.

Our findings showed a maximum age stage-specific survival rate (*s*_*xj*_) in control and *B. bassiana* than *M. anisopliae* treatment. The higher mortality in *M. anisopliae* may be attributed to its higher pathogenicity as compared to *B. bassiana*. The lower net reproductive rate in *M. anisopliae* treated ladybird beetle may also be attributed to the pathogenicity factor as fewer numbers of larvae were able to reach adulthood as compared to *B. bassiana* and control. However, no significant difference was found in the values of *e*_*xj*_ and *v*_*xj*_ in the EPFs-infected population and control group. Similarly, *l*_*x*_, *f*_*xj*_, and *m*_*x*_ curves also directed similar survival rates and fecundity of beetle in both EPFs treatments and control. Our findings suggest that EPFs do not affect the biological parameters of the ladybird beetle, *C. septempunctata*. It could be the ability of coccinellid to detect and avoid the EPFs conidia in terms of adaptation that enhances the survival rate and ultimately their fitness. The non-target effect of different EPFs on natural enemies of insect pests has been reported by many researchers. Ramanajum et al.^59^ reported that *B. bassiana* is safer for *C. septempunctata* and effectively control *Brevicoryne brassicae* (L.) under field conditions. Ullah et al.^60^ tested the virulence of *Isaria fumosorosea* and *B. bassiana*, against reduviid predator, *Rhynocoris marginates* (Heterop., Reduviidae) and reported no significant impact upon predation and survival rate of this biological control agent. Huang et al.^61^ reported that different concentrations of *B. bassiana* had no significant effect on biological parameters of a coccinellid, *Prynocaria cogener* (billberg). Similarly, different laboratory investigations documented that *B. bassiana* is not pathogenic to different beneficial arthropods. For example, *B. bassiana* was not harmful to *A. mellifera* L., *C. rufrilabris* Burmeister, *O. insidiosus* Say, *H. convergens* Gue´rin-Me´neville, *H. axyridis* (Pallas), *C. maculata* De Geer^62^. Harwood et al.^63^ studied the impact of laboulbenialean fungus *H. virescens* on coccinellids and recorded very low infection (< 5%).

However, laboratory-reared insects are more susceptible to infection by pathogens^64^. To explain the low incidence of EPFs infecting coccinellids under natural field conditions, the behavioral responses of the predators should be taken into consideration. According to Ormond et al.^52^ both male and female adult *C. septempunctata* avoid *B. bassiana* through contact with leaf surfaces and soil inoculations. Combined use of endophytic EPFs and entomophagous insects described low risks for predators and parasitoids in aphid IPM programs^65^. Use of *M. anisopliae* with *Nabis pseudoferus* (Hem., Nabidae) was reported a useful combination in controlling tomato leaf miner^66^. Similarly, Ríos-Moreno et al.^67^ documented that the *M. brunneum* is effective for the suppression of *S. littoralis* population and has a very low risk to the predator *C. carnea*.

The age-stage two-sex life table theory is a useful tool that allows the description of stage differentiation of insects and helps to create a comprehensive life table showing the demographic features of insect populations^40^. Our findings also demonstrated that the projection populations of coccinellid beetle were almost consistent in control and EPFs treatment*.* The projection of insect population growth using life table data is a vital tool in pest management and decision-making. Based on the findings of this study, it is suggested that EPFs are eco-friendly and don’t have any negative effect on coccinellid predators, the environment, and human health. Thus, these EPFs can be recommended to farmers to control aphids as an alternative to synthetic insecticides.

## Conclusion

The application of *B. bassiana* and *M. anisopliae* did not affect the generalist predator, *C. septempunctata*. Biological control agents and microbial pesticides are two such tools that can be used simultaneously due to their effectiveness, less risk to the environment and human health, and especially their potential compatibility. Our results indicate that commercial formulations of both EPFs are safer for coccinellid predators, suggesting these EPFs as compatible with other biological control agents (predator) and can be used in integrated pest management programs.

## Supplementary Information

Supplementary Tables.

## Data Availability

The datasets generated during and/or analysed during the current study are available from the first author on reasonable request.
