# Supplementary material for: Nondetrimental impact of two concomitant entomopathogenic fungi on life history parameters of a generalist predator, Coccinella septempunctata (Coleoptera: Coccinellidae)
Source: Sci Rep. 2021 Oct 19;11:20699. doi: 10.1038/s41598-021-00037-8 (PMC8526579; doi:10.1038/s41598-021-00037-8)
Supplement: Supplementary file 1 — Supplementary Tables. [file 41598_2021_37_MOESM1_ESM.doc]

Nondetrimental impacts of two concomitant entomopathogenic fungi on life history parameters of a generalist predator, *Coccinella septempunctata* (Coleoptera: Coccinellidae)

***P*-value of paired bootstrap test**

**Table S1**

**Paired bootstrap test of developmental time, adult longevity, fecundity, adult preoviposition period (APOP), total preoviposition period (TPOP), and oviposition days of *Coccinella septempunctata***

| Parameters | Stage | P value | | |
| --- | --- | --- | --- | --- |
|  |  | Control to BB | Control to MA | BB to MA |
| Developmental time (days) | Egg | 0.5795 | 0.4667 | 1.0000 |
| Larva | L1 | 1.0000 | 0.6473 | 0.6500 |
|  | L2 | 0.8232 | 0.5066 | 0.6642 |
|  | L3 | 0.8199 | 0.4932 | 0.6537 |
|  | L4 | 1.0000 | 0.6034 | 0.6073 |
| Pupa |  | 0.9257 | 0.3060 | 0.4251 |
| Adult longevity |  | 0.8965 | 0.4647 | 0.5464 |
|  | Male | 0.8393 | 0.8069 | 0.9842 |
|  | Female | 0.9874 | 0.9855 | 0.9985 |
| APOP (days) |  | 0.5886 | 0.5458 | 0.9730 |
| TPOP (days) |  | 0.6354 | 0.0739 | 0.2058 |
| Fecundity (eggs) |  | 0.9296 | 0.8903 | 0.9711 |
| Oviposition days |  | 0.5351 | 0.6848 | 0.9198 |

BB = *Beauvaria bassiana*, MA = *Metarhizium anisopliae*

**Table S2 Paired bootstrap test of net reproductive rate (*R_0_*), the intrinsic rate of increase (*r*), finite rate of increase (*λ*), generation time (*T*) and fecundity of *Coccinella septempunctata***

| Parameters | P value | | |
| --- | --- | --- | --- |
|  | Control to BB | Control to MA | BB to MA |
| *R_0_* | 0.0358 | 0.6791 | 0.0407 |
| *r* | 0.9975 | 0.9495 | 0.9500 |
| *λ* | 0.9994 | 0.9872 | 0.9882 |
| *T* | 0.9334 | 0.1895 | 0.2210 |
| Fecundity (eggs) | 0.9296 | 0.8903 | 0.9711 |

BB = *Beauvaria bassiana*, MA = *Metarhizium anisopliae*
